# LATIC–A linguistic analyzer for text and item characteristics

**DOI:** 10.1371/journal.pone.0277250

**Published:** 2022-11-03

**Authors:** Nadine Cruz Neri, Florian Klückmann, Jan Retelsdorf

**Affiliations:** Faculty of Education, Educational Psychology, University of Hamburg, Hamburg, Germany; European Commission, ITALY

## Abstract

Analyzing texts and items regarding their linguistic features might be important for researchers to investigate the effects of the linguistic presentation as well as for practitioners to estimate the readability of a text or an item. The Linguistic Analyzer for Text and Item Characteristics (LATIC) is a software that enables users to analyze texts and items more efficiently. LATIC offers a multitude of features at three different reading levels and can be used for texts and items in four different languages: English, French, German, and Spanish. It is open source, free to use and designed to be user-friendly. In this study, we investigated LATIC’s performance: LATIC achieves highly accurate results, while being extremely time saving compared to human raters. While developing LATIC, the respective features are tested continuously to ensure a high accuracy of results in the future.

## Introduction

In the last few decades, many tools have been developed to analyze text and item characteristics regarding their linguistic and syntactical complexity. To name but a few, the tools TAACO (Tool for the Automatic Analysis of Cohesion) [1] and ReaderBench [2] are available for English texts and items. However, tools to date all have their own strengths, but might also have a few shortcomings regarding their features and accessibility for researchers and practitioners. For instance, some tools only allow the analysis of texts and items in one language (e.g., ARTE [Automatic Readability Tool for English] [3]) or fail to report accuracy measures of the implemented features (e.g., RATTE [Regensburg Analysis Tool for Texts] [4]).

This article introduces a new tool that tries to overcome some of these shortcomings: A Linguistic Analyzer for Text and Item Characteristics (LATIC). LATIC enables users to analyze texts and items automatically at three reading levels: the word, sentence, and text level. Using the Stanford CoreNLP [5], LATIC annotates parts of speech (e.g., nouns, verbs) and counts them. Additionally, LATIC can calculate different traditional readability indices (e.g., Flesch Reading Ease [6]; Läsbarhetsindex [LIX] [7]) and other objective measures (e.g., average sentence length, word count). To date, LATIC is available for English, French, German, and Spanish and can be used on most operating systems without a registration or even an installation being needed.

In this article, we aim to introduce LATIC and highlight its features. First, we illustrate why it is important to analyze linguistic text and item characteristics and how LATIC can help users in doing so. Therefore, we show how LATIC can be used and which features LATIC (version 1.2.2) provides to date. Second, we investigate LATIC’s performance in terms of accuracy and time savings (compared to human raters). Finally, in the discussion we elaborate on advantages as well as limitations of LATIC comparing it to other tools.

### The importance of analyzing linguistic text and item characteristics

It is widely known that reading processes depend on reader characteristics, such as reading skills [8–10], but also on text and item characteristics [11, 12]. Regarding the latter, especially the linguistic complexity has gained a lot of attention in the past decades [13, 14]. In order to ensure that readers are able to comprehend written texts and items, it is essential to consider how linguistically complex these texts and items are. Many researchers and educators rely on traditional readability indices [15], such as the Flesch Reading Ease [6] and the SMOG (Simple Measure of Gobbledygook) [16] to estimate the complexity of a text or an item. However, it is argued that these readability indices might not be appropriate predictors of linguistic complexity since they are usually based on a few variables only, such as word and sentence length [17]. In fact, it is known that text and item characteristics that are not implemented into these readability indices can also generate difficulties in comprehension. The number of prepositional phrases, for instance, hinders comprehension and is associated with lower performance of students [18] and adults [12]. Hence, it is important not to rely on traditional readability indices only [17], but to analyze texts and items in more depth.

Many researchers draw on secondary analyses of test items and examine how specific text and item characteristics are linked to individuals’ performance on those test items [12, 18]. For this, it is necessary to analyze said texts and items as a first step. Text and item characteristics can be manually tagged, coded and counted. For instance, Shaftel et al. [19] created a coding list for raters in order to rate test items regarding 17 characteristics. However, this procedure might be error prone and time consuming. Thus, there are many natural language processing (NLP; see [20] for a detailed description) tools that automatize this process in order to save resources [21]. There are many tools researchers and practitioners can choose from depending on the linguistic analyses they are aiming for. For instance, there are many (online) tools for parts of speech tagging, such as the online demo version of the Stanford CoreNLP [22]. In case users are interested in readability indices, they can opt for ARTE [3], when calculating English texts and items or RATTE [4], when analyzing German texts or items. Users are even confronted with more tools to choose from, when they want to analyze rather objective text and item characteristics, such as sentence length or word count (e.g., Coh-Metrix [23]; the SiNLP [Simple Natural Language Processing Tool] [21]).

However, it might be time consuming having to use several tools at once, which all might have their own strengths but also shortcomings. For instance, most tools are only available in one language [1, 3, 23, 24]. More importantly, to the best of our knowledge, several developers provide no or only limited accuracy measures for the individual features implemented in their tools [4, 21], which makes it difficult to decide whether the results are reliable or not. With LATIC, we aim to provide users with a tool that resolves some of the shortcomings other tools have. In the following, we introduce our tool and investigate how accurate LATIC’s results are compared to human ratings. In the discussion, we will further compare LATIC’s features with other tools, highlighting advantages as well as limitations of the tool.

## The software LATIC

### Technical details

LATIC is a java application that is free and open source. LATIC can be used on Windows and Linux (e.g., Debian, Ubuntu) operating systems as well as on macOS to analyze texts and items. The duration of text and item analyses depends on the performance of the computer and the extent of the analyses. In our test runs, the analyses usually took a few seconds only.

For some features (i.e. tagging parts of speech), LATIC relies on a NLP tool. For LATIC, we used a NLP tool named Stanford CoreNLP [5]. The Stanford CoreNLP [25] is Java application providing users with several linguistic annotations for any kind of text, including parts of speech tagging. We always incorporate the latest version of the Stanford CoreNLP [5]. When submitting this manuscript, version 4.4.0 was the newest.

#### How to use LATIC

LATIC is an intuitive and easy to use software that can be downloaded on https://download.latic.software. Users are able to use the software after installing LATIC (a step-by-step instruction is provided with the download). However, if users do not want to install LATIC, they can start it via the console. With using LATIC, data security is guaranteed since all analyses are performed on users’ local computers.

**Fig 1:**
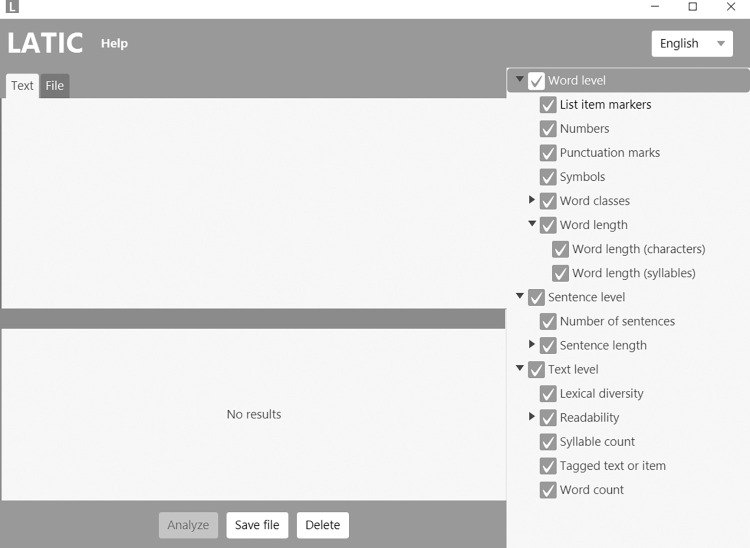
User Interface of LATIC (Version 1.2.2).

Fig 1 depicts the user interface of LATIC (version 1.2.2). In order to analyze a text or an item, users first need to choose the language of the text or item on the upper right. Second, users must enter the text or item in the text field or upload a document containing the text or item. Third, the users choose the characteristics that shall be analyzed on the right. Finally, the users need to click on the “Analyze” button to display the results. This step might take a few seconds. If users want to save their results, they can do so by clicking on the “Save file” button and choosing an appropriate file format, such as.csv files. The “Delete” button enables users to delete the results. A more detailed example is uploaded on GitHub [26]: It demonstrates the analysis of a famous tale and includes step-by-step instructions (including screenshots) to obtain the results.

If users need help, they can click on the “Help” button on the top row. They are provided with the option to write an e-mail or to retrieve the documentation of the software [27], including step-by-step instructions and detailed information about every feature of LATIC (see next section).

In order to ensure correct results, users need to keep a few factors in mind. First, similar to other software, it is essential to use correct spelling for reliable results, especially with regard to the parts of speech tagging. Second, abbreviations and measuring units should be avoided since they tend to bias both the tagging and the calculation of item characteristics (e.g., readability indices, average word length). Third, cardinal numbers should be written as Arabic numbers if tagging parts of speech is relevant for the analysis of the item. To calculate other characteristics, such as the syllable count or a readability index, the cardinal numbers should be spelled out. Finally, it is important to note that the Stanford CoreNLP [5] tags every juxtaposition of characters separated by spaces or punctuation marks individually. Hence, for instance, *Red Sea* would be counted as two words, each getting the tag of a proper noun.

### Features

LATIC allows users to analyze texts and items regarding their characteristics at different levels, i.e., the word, sentence and text level. Table 1 includes all features of LATIC. Due to the amount of features that LATIC provides, going into detail for every single features, would go beyond the scope of this paper. In the documentation of LATIC [27], we present every single feature of LATIC in much more detail, including short explanations and examples.

**Table 1:** Overview of LATIC’s features.

Level	Text and item characteristics	Available in			
		English	French	German	Spanish
Word	Adjectives	✓	✓	✓	✓
	Adpositions		✓	✓	✓
	Adverbs	✓	✓	✓	✓
	Cardinal numbers	✓	✓	✓	✓
	Conjunctions	✓	✓	✓	✓
	Determiners	✓	✓	✓	✓
	Existential there	✓			
	Infinitive *to*	✓			
	Interjections (only primary)	✓	✓	✓	✓
	List item markers	✓			
	Modals	✓	✓	✓	✓
	Nouns	✓	✓	✓	✓
	Particles	✓		✓	✓
	Possessive endings	✓			
	Prepositions	✓			
	Pronouns	✓	✓	✓	✓
	Proper nouns	✓	✓	✓	✓
	Punctuation marks	✓	✓	✓	✓
	Symbols	✓	✓	✓	✓
	Tenses	✓			
	Unknown or uncertain	✓		✓	✓
	Verbs	✓	✓	✓	✓
	Word length (characters)	✓	✓	✓	✓
	Word length (syllables)	✓		✓	
Sentence	Number of sentences	✓	✓	✓	✓
	Sentence length (characters w/o spaces)	✓	✓	✓	✓
	Sentence length (characters w/ spaces)	✓	✓	✓	✓
	Sentence length (syllables)	✓		✓	
	Sentence length (words)	✓	✓	✓	✓
Text	Automated readability index	✓			
	Coleman-Liau index	✓			
	Flesch-Kincaid grade level	✓			
	Flesch reading ease	✓		✓	
	gSMOG			✓	
	Gunning Fog index	✓			
	Lexical diversity	✓	✓	✓	✓
	LIX	✓	✓	✓	✓
	SMOG	✓			
	Syllable count	✓		✓	
	Tagging the whole item	✓	✓	✓	✓
	Wiener Sachtextformel			✓	
	Word count	✓	✓	✓	✓

First, LATIC enables the users to tag and count different parts of speech contained in a text or an item. The tagging of parts of speech is mostly based on the Stanford CoreNLP [5]. The only exceptions are the taggings of primary interjections and symbols in the French, German, and Spanish versions due to frequent errors in our test runs; these taggings are made by LATIC. The annotation in English is based on the Penn Treebank Tagset [28]; for French, German and Spanish the UD (version 2) tagset is used [29].

Furthermore, LATIC counts up the tags, thus providing the user with an absolute frequency of the parts of speech chosen for analysis. LATIC is also able to display the tags for every word or rather character juxtaposition. This feature enables the user to examine the tags given out by the Stanford CoreNLP [5].

Second, LATIC enables the user to analyze texts and items regarding other objective characteristics at all three levels, such as the average word and sentence length (see Table 1).

Third, LATIC’s features include the calculation of several readability indices (see Table 1). As noted above, traditional readability indices are heavily criticized [17] and we mostly agree with the critique. However, since the calculation of readability indices can be helpful for some research questions [15], we want to enable users to calculate them more easily. One must note that not every readability index is available in all languages because readability indices are not necessarily transferrable to other languages [30]. For instance, the Flesch Reading Ease [6] and the SMOG [16] for English texts and items needed to be adapted for the German language, resulting in the Flesch index [31] and the gSMOG [32]. Hence, we only implemented readability indices suitable for the respective languages.

### The present study

In this study, we aim to first examine the performance of LATIC in terms of accuracy as well as time saving compared to analyses performed by human raters. For this, we aimed to focus on three aspects. During the development of LATIC, the implemented features were repeatedly tested and bugs were fixed immediately when noticed. However, there were two exceptions: The part of speech tagging by means of the Stanford CoreNLP 4.4.0 [5] as well as the syllable count. No errors occurred during our multiple test runs regarding all other features (such the counting of parts of speech and the calculation of text and item characteristics). Thus, we particularly wanted to investigate the accuracy of (1) the parts of speech tagging and (2) the syllable count. Furthermore, we aimed to investigate how the performance of LATIC compares to humans’ performance in terms of accuracy as well as time. Examining these aspects, we focus on the English and German language.

## Method

### Parts of speech tagging

In order to test the accuracy of the tagging provided by the Stanford CoreNLP 4.4.0 [5], we compared the tokens given out by LATIC with the tokens of two well-known corpora (see [27] for more details). For the English language, we used the MULTEXT-East 4.0 corpus [33]. The corpus was manually tagged and consists of the book “1984” by George Orwell [34]. For the German language, we used the TIGER corpus 2.2 [35]. The corpus was tagged semi-automatically and consists of newspaper articles [36].

For evaluating the accuracy, we used around 10,000 tokens of each corpus. These tokens were analyzed with LATIC. However, to compare the accuracy of LATIC in a reasonable way, we needed to take a few steps. First, we deleted all words that were misspelled since a right spelling of words is essential for tagging the parts of speech correctly. Second, we matched the respective tagsets to the ones used by the Stanford CoreNLP 4.4.0 [5]. Pronouns, for instance, are tagged in much more detail in the MULTEXT-East 4.0 corpus [37] than in the tagsets used by the Stanford CoreNLP [5]. Third, we examined how many tokens were tagged correctly by LATIC by means of the Stanford CoreNLP 4.4.0 [5].

### Calculation of syllable count

In the English language, we used a script [38] to count the syllables. First, we tested the script with *N* = 9,107 common words (https://en-syllables.latic.software/). In a second step, we optimized the results by adapting the script: We created a list of words that were counted incorrectly and implemented the correct syllable count for each of those words [39].

In the German language, we created an algorithm to count the syllables. First, we tested this algorithm with *N* = 9,524 common words according to the Leipzig Corpora Collection [40]. Again, in a second step, we optimized the algorithm by including (1) additional rules to correctly count the syllables, (2) a list of words, in which the syllables were not correctly counted, and (3) Anglicisms and Gallicisms that are common in German.

### Comparison with human performance

We chose five openly available science items that were used in the PISA study (Programme for International Student Assessment) in 2015 [41]: (1) the introductory text of item S656, (2) S656Q01, (3) S656Q02 as well as (4) the introductory text of item S641, and (5) the item S641Q01. The chosen items ranged from 22 to 101 words and did not include any illustrations.

We asked four people to analyze the items regarding the selected text and item characteristics. The four raters were two student assistants with little experience in linguistic coding, one foreign language assistant with decent experience in linguistic coding as well as the first author of this paper who has a lot of experience in linguistic coding. The first three raters were given an instruction on how to analyze text and item characteristics by the first author. Especially the two student assistants received further explanations and support due to their little experience in coding.

We randomly selected thirteen text and item characteristics that the raters should analyze (see Table 2). The raters were asked to (1) give their ratings regarding the characteristics, (2) state how confident they were in their results (ranging from 1 [not at all] to 5 [very confident]), and (3) to measure how long it took for them to analyze each item.

**Table 2:** Comparison of coders’ ratings and LATIC’s results including interrater-reliability.

Ratings	Krippendorff’s α	Item 1		Item 2		Item 3		Item 4		Item 5		Total	
		Rater	LATIC	Rater	LATIC	Rater	LATIC	Rater	LATIC	Rater	LATIC	Rater	LATIC
Nouns	0.96	22.50 (1.00)	24.00	25.00 (0.82)	24.00	5.75 (0.50)	6.00	14.00 (2.00)	11.00	14.00 (2.00)	11.00	16.25 (7.16)	15.20 (8.29)
Adverbs	0.91	0.25 (0.50)	1.00	4.50 (1.00)	7.00	0 (0)	1.00	0.25 (0.50)	1.00	0 (0)	1.00	1.00 (1.86)	2.20 (2.68)
Pronouns	0.04	1.50 (1.00)	2.00	2.75 (2.50)	0	0.75 (0.50)	0	2.50 (1.73)	3.00	2.25 (0.96)	2.00	1.95 (1.54)	1.40 (1.34)
Verbs (total)	0.81	6.25 (1.89)	8.00	15.00 (2.94)	18.00	5.00 (0.82)	5.00	10.25 (1.26)	11.00	7.00 (0.82)	12.00	8.70 (4.00)	10.80 (4.87)
Verbs in past tense	0.72	0.50 (1.00)	0	5.50 (1.00)	6.00	0 (0)	0	1.00 (1.15)	2.00	1.00 (2.00)	0	1.60 (2.30)	1.60 (2.61)
Number of sentences	1.00	4.00 (0)	4.00	7.00 (0)	7.00	1.00 (0)	1.00	4.00 (0)	4.00	6.00 (0)	6.00	4.40 (2.11)	4.40 (2.30)
Syllable count	0.99	96.00 (1.15)	95.00	168.25 (2.87)	167.00	36.25 (1.26)	36.00	63.00 (2.94)	66.00	77.00 (6.22)	80.00	88.10 (45.82)	88.80 (48.83)
Word count	1.00	60.50 (0.58)	61.00	101.00 (0)	101.00	22.00 (0)	22.00	43.75 (1.5)	43.00	53.00 (0)	53.00	56.05 (26.61)	56.00 (29.09)
Word length (characters)	0.97	5.36 (0.06)	5.30	5.02 (0.04)	5.01	5.26 (0.40)	5.23	4.84 (0.11)	4.91	4.30 (0.07)	4.32	4.97 (0.37)	4.95 (0.39)
Sentence length (syllables)	0.99	24.00 (0.29)	25.25	24.04 (0.41)	24.86	36.25 (1.26)	39.00	15.75 (0.74)	17.75	12.83 (1.04)	14.50	22.57 (8.40)	24.27 (9.44)
Sentence length (words)	0.11	31.63 (32.91)	15.25	14.43 (<0.01)	14.43	22.00 (0)	22.00	10.94 (0.38)	10.75	8.83 (<0.01)	8.83	17.56 (15.63)	14.25 (5.07)
Lexical diversity	0.96	0.69 (0.05)	0.72	0.56 (0.01)	0.57	0.86 (<0.01)	0.91	0.75 (0.02)	0.77	0.58 (<0.01)	0.58	0.67 (0.12)	0.71 (0.14)
LIX	0.11	35.84 (23.48)	46.40	38.14 (0.10)	38.19	44.72 (<0.01)	44.73	29.27 (1.91)	29.35	28.72 (1.84)	27.70	35.34 (11.20)	37.22 (8.51)

### Ethical approval

An ethics committee approval was not needed due to solely working with entered text or items in LATIC. Sensitive data, such as from vulnerable people, were not used in developing the software or preparing this manuscript. All four raters of legal age gave their consent in participating in the study.

## Results

### Parts of speech tagging

Regarding the plain comparison of tokens, LATIC correctly tagged *n* = 8,651 out of all *N* = 9,989 (86.61%) in the English language, and *n* = 9,093 out of all *N* = 9,997 (90.96%) in the German language. As noted above, some modifications needed to be made to ensure a reasonable comparison of the tokens. After these modifications, *n* = 9,048 out of the remaining *N* = 9,755 (92.75%) of the tokens were correctly tagged by the Stanford CoreNLP 4.4.0 [5] in the English language; in the German language, *n* = 9,271 out of the remaining *N* = 9,879 (93.85%) of the tokens were correct. Finally, we analyzed the most frequent errors in tagging by examining the first 5,000 tokens. In both languages, the differentiation between determiners and pronouns seemed to be the most challenging (English: 37.84% of all errors; German: 21.74% of all errors). However, this was to be expected since the differentiation between these two word classes is not always clear [29]. In the English language, the distinction of adjectives and the tagging of (1) prepositions and subordinating conjunctions (11.28% of all errors), and (2) all types of verbs (8.52% of all errors) were by far the second and third most frequent errors. In the German language, the second and third most frequent errors were the distinction between nouns and proper nouns (13.71% of all errors) and the distinction between adjectives and adverbs (9.36% of all errors).

### Calculation of syllable count

In the English language, the syllables of *n* = 8,723 words (95.78%) were counted correctly. After adapting the script by Wormer (2021), the syllables of all *N* = 9,107 test words (100%) were correctly counted. In the German language, the syllable count of *n* = 9,190 words (96.49%) was correctly calculated. After optimizing our algorithm, the syllable count was correct for *n* = 9,522 test words (99.98%).

### Comparison with human performance

All results by the four raters as well as LATIC’s results are openly available on https://rating-results.latic.software/. The means of the raters’ ratings as well as LATIC’s results are depicted in Table 2. In terms of accuracy, the raters felt confident that their ratings were accurate (*M* = 4.27, *SD* = 0.55). In fact, conducting a Mann-Whitney-U-test, there are no significant differences between the coders’ and LATIC’s results, *U* = 2081.50, *p* = .885. We also tested whether the overall ratings of each individual rater differed from LATIC’s results by conducting further Mann-Whitney-U-tests. All differences were non-significant (*p* > .570).

Despite the differences not being significant, LATIC still achieves higher accuracy than the raters regarding certain text and item characteristics, such as calculating the lexical diversity [42] and the readability index LIX [7]. This can be mostly traced back to human raters’ calculation errors, which LATIC as a technical software does not make.

The interrater-reliability (Krippendorff’s α) [43] varies tremendously between the different text and item characteristics. It can be positively mentioned that the raters reach (very) good reliabilities (Krippendorff’s α ≥ 0.80) for more than half of the text and item characteristics (e.g., number of sentences, word count). However, it becomes apparent that not all ratings made by humans are suitable to work with since some interrater reliabilities are very low (e.g., pronouns, LIX).

In total, the four raters needed *M* = 4,374.80 seconds (~ 72 minutes; *SD* = 1,846.25) to analyze all five items regarding the thirteen text and item characteristics. For the analyses of each item, the four raters used *M* = 2.60 (*SD* = 0.55) different tools, which they used *M* = 7.80 (*SD* = 0.45) times. The first author further analyzed the five items by means of LATIC and finished the analyses within 80 seconds, including starting the software and choosing the text and item characteristics on the right sidebar (see section “How to Use LATIC”).

## Discussion

As noted above, there are a few tools and web applications used by researchers to automatize the process of analyzing text and item characteristics. However, these tools and applications might only be partially suitable for envisioned purposes. Thus, in this article, we introduced the new tool LATIC which combines many linguistic features, such as parts of speech tagging, the calculation of objective text and item characteristics as well as readability indices. On the basis of the Stanford CoreNLP [5], users can analyze texts and items in English, French, German, and Spanish. Drawing on our results, LATIC achieves very high performance in terms of accuracy as well as time saving compared to human raters. Thus, LATIC provides reliable results, while also saving users’ resources.

### Strengths of LATIC

In research, it is important to work with reliable results in order to draw plausible and accurate conclusions. Thus, it is essential to work with tools and software that provide researchers with such reliable results. Unfortunately, to the best of our knowledge, many authors also fail to provide measurements of accuracy for their tools or rather for specific features of their tools (for example [4]). Our article shows that users can analyze linguistic text and item characteristics with high accuracy in a short time with LATIC. The tagging provided in LATIC by means of the Stanford CoreNLP 4.4.0 [5] reaches very good accuracy in our analyses, which corroborates prior research. In other evaluation studies, the Stanford CoreNLP [5] usually reaches one of the best accuracy rates compared to other parts of speech taggers [44, 45]. Furthermore, the accuracy of the syllable count is also high, especially when adapting the script and algorithm to optimize the syllable count feature. Finally, comparing LATIC’s performance with human raters, the results of our analyses showed that LATIC reaches similar accuracy as human raters. At this point, it is important to note that the interrater-reliability varies tremendously for some linguistic text and item characteristics suggesting that a tool like LATIC might provide more adequate rating results than human raters. In sum, LATIC provides reliable results while saving the users valuable resources, such as time and effort, since the analyses usually only takes (milli-) seconds.

In terms of scientific aspects, many authors traditionally tend to manually tag, code and count text and item characteristics, such as word count [9] or prepositions [19]. While doing this, the human raters might fall back on several different tools, which was the case in our study. This might be due to some web applications offering only a limited number of features (e.g., T.E.R.A [46]) or even just one (e.g., LIX calculator [47]). LATIC provides the users with the advantage of combining many features in one software that are essential for evaluating the readability of texts and items. As noted above, LATIC as a tool offers users to automatically tag and count parts of speech. Furthermore, LATIC calculates further characteristics as well as traditional readability indices, which are regularly used in research [10, 19, 48].

In terms of breadth of features, most tools and applications only analyze texts or items in one specific language (e.g., ARTE [3]). LATIC offers the linguistic analyses of texts and items in four languages (English, French, German, and Spanish). Further languages can be implemented into LATIC, if suitable natural language processing tools are available. For instance, besides the languages already implemented into LATIC, the Stanford CoreNLP further supports tagging parts of speech in the Arabic, Hungarian, Italian, and Chinese languages [25]. However, since none of the authors is fluent in neither of those languages, these could not be implemented (yet).

Regarding usability aspects, some web applications are not always available online (Coh-Metrix [46]; Stanford CoreNLP demo [22]), require a registration (e.g., CTAP [49]; Text Inspector [24]), or are not free to use (e.g., LIWC [50]; Text Inspector [24]). LATIC is open source and free to use. Thus, the code is openly available [39] and can be audited and modified. Furthermore, users can request features to be implemented into LATIC and may report bugs under https://github.com/florianklueckmann/LATIC/issues or by sending an e-mail to hello@latic.software. It is also of advantage that the interface is user-friendly and intuitively usable, thus diminishing long training periods. All features are documented in detail [27], which provides users with essential information on how the results of LATIC are formed. LATIC also enables users to work with the results by allowing users to save and import the results into statistical analysis software, if needed.

### Limitations

Despite the strengths of LATIC, a few limitations need to be discussed. First, in order to obtain the most reliable results possible, users need to follow a few instructions. Similar to other tools and software, this includes ensuring the correct spelling and avoiding abbreviations (see above). This means that users have a high responsibility to ensure the entered texts and items are examined and—if necessary—modified accordingly. This means that, for instance, texts written by students can only be analyzed regarding their characteristics, if the spelling is corrected beforehand. However, it might be the case that researchers or practitioners are especially interested in these errors. In this case, we would recommend using the software GAMET (Grammar and Mechanics Error Tool) [51]. The software enables users to locate structural as well as mechanical errors within an English text, which is the focal point of the tool. Parts of speech tagging or the calculation of text and item characteristics, however, cannot be executed.

Second, some linguistic characteristics prominent in readability research, such as cohesion [52] or phrases [18], cannot be analyzed in LATIC. However, there are a number of tools enabling users to analyze their English texts and items regarding cohesion. For instance, the tool TAACO [1] estimates over 150 indices of global and local cohesion. While TAACO provides many indices of cohesion, other text and item characteristics, such as readability indices or the syllable count, cannot be calculated. In case, users want to analyze phrases, we recommend the tool TAASSC (Tool for the Automatic Analysis of Syntactic Sophistication and Complexity) [53, 54]. While TAASC focuses on estimating the syntactic sophistication as well as complexity of English texts, parts of speech tagging as well as the calculation of the syllable count and readability indices are not available.

Finally, when developing LATIC, we decided to implement the Stanford CoreNLP [5] rather than other NLP tools due to its high accuracy rates in prior research [44, 45]. However, one must note that the Stanford CoreNLP [5] seems to perform relatively well on generic text and item types, but not in significantly different text types. For instance, other parts of speech taggers outperform the *Stanford* CoreNLP [5] in social media texts [55] or ReadMe documents of software [56]. The main intent was to provide educators and (readability) researchers the opportunity to analyze linguistic text and item characteristics with LATIC. Thus, we opted for an NLP tool that performed well on rather formal text and item types found, for instance, in the educational context.

## Conclusion

The analyses of linguistic text and item characteristics might not only be necessary for investigating research questions regarding language in texts and items, but also for practitioners, such as teachers and educators. Traditionally, texts and items are analyzed manually [9, 19], which is very time consuming. LATIC is a user friendly and free to use java application enabling users to analyze texts and items in four languages (English, French, German, and Spanish) regarding their linguistic characteristics. LATIC offers features at the word, sentence and text level including the tagging and counting of parts of speech and the calculating of traditional readability indices. All features were tested thoroughly and reach remarkable accuracy.
